# Chronic inflammation and asthma

**DOI:** 10.1016/j.mrfmmm.2009.09.005

**Published:** 2010-08-07

**Authors:** Jenna R. Murdoch, Clare M. Lloyd

**Affiliations:** Leukocyte Biology Section, National Heart and Lung Institute, Faculty of Medicine, Imperial College London, London SW7 2AZ, UK

**Keywords:** Asthma, Chronic allergic airway inflammation, Remodelling

## Abstract

Allergic asthma is a complex and chronic inflammatory disorder which is associated with airway hyper-responsiveness and tissue remodelling of the airway structure. Although originally thought to be a Th2-driven inflammatory response to inhaled innocuous allergen, the immune response in asthma is now considered highly heterogeneous. There are now various *in vivo* systems which have been designed to examine the pathways leading to the development of this chronic immune response and reflect, in part this heterogeneity. Furthermore, the emergence of endogenous immunoregulatory pathways and active pro-resolving mediators hold great potential for future therapeutic intervention. In this review, the key cellular and molecular mediators relating to chronic allergic airway disease are discussed, as well as emerging players in the regulation of chronic allergic inflammation.

## 1 Asthma

Allergic asthma is a heterogeneous disorder of the conducting airways involving chronic airway inflammation, declining airway function and tissue remodelling. The prevalence of asthma has rapidly increased over the last few decades to epidemic proportions and there are now an estimated 300 million sufferers worldwide, a total that is expected to rise dramatically over the next 15–20 years [1]. Asthma is associated with enormous healthcare expenditures and, despite the advances in effective therapy, the consequential economic burden associated with disease control and morbidity continues to escalate [2].

**Fig. 1:**
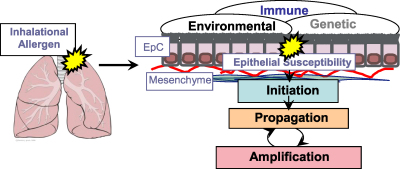
Gene environment interactions in asthma. Asthma is an inflammatory disorder of profound heterogeneity with strong genetic and environmental components. Local airway susceptibility factors together with allergen-specific immune polarisation interact both in the induction and subsequent expression of the disease phenotype. *Key*: EpC, epithelial cell.

Asthma is thought to arise from the complex interplay of genetic susceptibility and environmental influences, such as timing and dose of allergen and co-exposure to infection [3]. This culminates in an inappropriate inflammatory response led by the T-helper type (Th)-2 lymphocytes to normally harmless airborne allergens [4] (summarised in Fig. 1). Although no specific gene or environmental factor solely accounts for asthma, the genetic predisposition to mount a local mucosal immunoglobulin (Ig) type E (IgE) response, known as atopy, is one of the strongest risk factors for developing the disease [3,5]. The majority of asthma is associated with atopy [6], however there are also clinically defined variant forms of the disorder which are independent of atopy, i.e. do not mount an IgE response to environmental allergens [7]. Th2 associated inflammation and IgE production are also features of non-atopic or intrinsic asthma, although what drives this process remains unknown [8]. Indeed, asthma is now considered a heterogeneous disorder comprised of distinct endotypes [9,10]. This review will provide an overview of the immunopathological mechanisms underlying the various clinical features of asthma as well as some important remaining questions in asthma research.

### 1.1 Airway hyper-responsiveness

Clinically, asthma presents as a physiological dysfunction of the lung characterised by breathlessness, wheeze and a variable airflow obstruction [11]. This accounts for the dramatic increase in responsiveness of the conducting airways known as airway hyper-responsiveness (AHR) [12]. AHR, considered the hallmark of the asthma phenotype, is defined as the ease with which the airways narrow in response to a bronchoconstrictive challenge and manifests as a combination of increased sensitivity and reactivity for a given stimulus [13]. AHR is a fundamental abnormality in asthma however at present there is no clear or consistent association between immunological and structural features of asthma and the increased responsiveness of the airways. Rapid bronchiolar constriction and reversal with bronchodilators suggests that ASM is involved. Increased ASM is seen in kids and removal of ASM by thermoplasty, improves AHR demonstrating a contribution of ASM to AHR [14]. Airway wall thickening due to remodelling is associated with increased AHR [15]. However, Niimi et al. demonstrated an inverse relationship between AHR and airway wall thickness [16]. This is discussed fully in recent review [17]. Recent studies have demonstrated a variety of cellular inflammatory phenotypes associated with asthma. An eosinophilic or neutrophilic infiltrate is a common feature of allergic airway inflammation and this has been correlated clinically with AHR although similar results were not found in murine studies. Increased presence of mast cells [18] and Th2 [19] cells also correlates with AHR. Understanding the mechanisms underlying this response is of great importance.

## 2 Immunopathogenesis of chronic asthma

### 2.1 Acute episodes, persistent inflammation and airway remodelling

Inflammation is a response of the immune system to injury which is beneficial to the host under normal circumstances. However, an aberrant immune response to non-pathogenic stimuli in the asthmatic airway leads to a chronic inflammatory response relevant to the pathogenesis of the disease [20]. Episodes of acute inflammatory reactions are often accompanied by an underlying chronic inflammation even in the absence of continuous allergen exposure [11]. The complex interplay between the multi-cellular inflammatory infiltrate and parenchymal lung tissue cells is orchestrated by a broad network of self-amplifying bioactive mediators, including cytokines, antibodies and growth factors [21]. Inflammatory leukocyte recruitment is directed by small inflammatory soluble molecules known as chemokines [21,22]. Inappropriate immune activation is thought to be, in part, responsible for the chronicity of allergic asthma, however there is now increasing evidence that dysregulation of endogenous immune regulating processes are, in part, also responsible for the development of this disease [23].

The immune system has classically been divided into two major arms: innate and adaptive. The evolutionarily primitive innate immune system forms an immunosurveillance network in the periphery to initiate an immediate beneficial response to foreign agents [24]. Adaptive immune lymphocytes re-circulate within the blood and lymphatic system and have the ability to mount an antigen-specific immune response accompanied by development of long lasting memory to subsequent challenges by the same antigen [25]. The potency of the adaptive immune systems lies in its ability to generate billions of different antigen receptors from multiple gene segments [26]. The innate and adaptive immune systems are closely interlinked since the innate immune response defines the phenotype of subsequent antigen-specific adaptive immunity [25]. Optimal immune function relies on contributions from both divisions in equilibrium with immunoregulatory components [27]. Allergic inflammation is mediated by cells from both the innate, adaptive and surrounding tissue cells. Recently, however, the distinctions between innate and adaptive immunity have become blurred. Indeed, certain leukocytes populations such as γδT cells and NKT cells do not fit either convention [28]. An overview of the various immune leukocytes involved in the allergic airway is discussed below.

## 3 Allergic sensitisation

### 3.1 Th2 priming by DCs

It was long enigmatic how sensitisation to natural allergens occurred since, unlike pathogen-associated antigens, not all allergens are intrinsically immunogenic. One of the earliest steps in the establishment of allergic sensitisation is the generation of an antigen-specific T cell response to an allergen [29]. Th2 responses are typical of allergic asthma; however, T cells do not react directly to inhaled antigens and require critical instructions provided the innate immune system. Dendritic cells (DCs) are considered the most powerful antigen-presenting cells (APCs) and form an innate immune network within the lung tissue [30]. They express a variety of evolutionarily ancient germline-encoded receptor families, known as pattern-recognition receptors (PRRs) which enable constant monitoring of the airways. PRRs recognise a broad range of invariant structures such as microbial components, known as pathogen-associated molecular patterns (PAMPs) which enable the immune system to detect pathogens and promote elimination [31]. Many allergens are contaminated with agonists of PRR enabling them to induce DC “maturation”. This involves up-regulation of co-stimulatory molecules and acquisition of the capacity to migrate to the draining lymph nodes, enabling them to interact with an effectively prime an antigen-specific effector T cell response [32–34]. Recently, generation of allergic Th2 responses to allergen have been demonstrated via PRR such as Toll-like receptors (TLRs) suggesting that this may be a critical component of sensitisation to some allergens [35]. However, not all allergens are intrinsically immunogenic or have PRR agonistic potential. Danger signals or damage-associated molecular patters (DAMPs) in the absence of infection can also influence dendritic cell activation [32,36]. Many allergens contain enzymatic activity which liberate DAMPs in the lung microenvironment [37]. Indeed, Hammad et al. have shown that triggering of epithelial expressed TLR-4, a receptor that recognises conserved components of microbes, helps drive the development of allergic reactions to a common household antigen [38]. In addition, the release of Uric acid by dying cells can induce Th2 polarisation and atopic asthma via triggering of NALP3 inflammasome in DCs [39].

There is now compelling evidence in support of the crucial role played by DC in determining the functional outcome of T cells responses following allergen exposure and inception of atopic asthma [30]. In addition to T cell priming, it is now well established that DCs are also essential for maintenance of the Th2 response towards aeroallergens [40]. It is important to note that, in addition to conventional DCs (cDCs), an additional DC subset known as plasmacytoid (pDC) are present in the lung [41]. Interestingly, transfer of allergen loaded pDC suppressed inflammation in allergen sensitised and challenged mice, whereas depletion of pDC abrogated this tolerance [34], and as such pDCs are thought to have a regulatory influence in the airway. Manipulation of DC function can have profound effects on the allergic inflammatory response highlighting these cells as potential targets for therapeutic intervention. This has been excellently reviewed elsewhere [42].

**Fig. 2:**
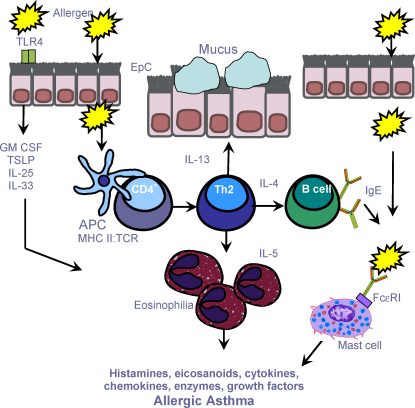
Immune cells and the inflammatory cascade in asthma. Initial exposure(s) to allergen leads to the activation of allergen-specific Th2 cells and IgE synthesis (sensitisation). Subsequent allergen exposures cause inflammatory-cell recruitment, activation and mediator release. IgE-sensitised mast cells expressing the high affinity IgE receptor (FcɛRI) degranulate, releasing both pre-formed and newly synthesized mediators including histamine, leukotrienes and cytokines, which promote vascular permeability, smooth muscle contraction and mucus production. Chemokines released by inflammatory and resident cells direct recruitment of inflammatory cells characterised eosinophils and Th2 cells. Eosinophils release an array of pro-inflammatory mediators, including leukotrienes and basic proteins and mediators such as, IL-5. *Key*: APC, antigen-presenting cell; ASM, airway smooth muscle; EpC, epithelial cell; GM-CSF, granulocyte monocyte colony stimulating factor; MHC, major histocompatibility; TCR, T cell receptor; TSLP, thymic stromal lymphopoietin.

Activation of Th2 cells by APCs leads to the production of cytokines such as IL-4. The presence of an IL-4 rich cytokine milieu promotes co-stimulatory interactions between T cells and B cells initiating class switching and secretory plasma B cell affinity maturation to produce IgE [43] (Fig. 2). However, priming an allergen-specific Th2 response from naïve CD4^+^T cells is dependent on exogenous IL-4 in the surrounding milieu [44]. The nature of the cells that produce ‘early’ IL-4 required for this differentiation remains unknown. Interestingly, basophils are potential candidate responsible for early production of this cytokine [45]. Furthermore, recent investigations have elegantly demonstrated the importance of allergen presentation by basophils to the development of allergic sensitisation *in vivo*, and the ability of basophils to initiate Th2 differentiation in the absence of DCs *in vitro* [46–48]. This information offers a new paradigm for the initiation of Th2 immunity which will no doubt lead to exciting developments about their function in disease and potential as a therapeutic target. Chronic inflammatory conditions encountered *in vivo* are thought to promote antigen-presenting capabilities in a variety of different cell types. Indeed many immune cells associated with the asthmatic phenotype demonstrate APC capabilities including γδT cells [49], eosinophils [50,51] and lung structural cells [52]. During chronic inflammation, it is thought the interface between T cells and non-professional APCs in the asthmatic lung and serves to further propagate inflammatory response [53].

The pulmonary epithelium is the first point of contact for inhaled environmental allergens and is increasingly implicated as a central player in the Th2 cell sensitisation process [54]. The relationship between the pulmonary innate immune system and surrounding tissue is essential to the proximal events leading to Th2 mediated allergen sensitivity and there is now a growing appreciation of the contribution of innate immune system to asthma [55,56]. Indeed, many products of asthma susceptibility genes have been linked to the innate immune system [4]. Epithelial cells influence DC function through direct cell–cell interaction and via the release of mediators [55]. Allergens such as house dust mite (HDM), can directly or indirectly interact with the innate immune functions of airway epithelial cells and DCs [38,57]. Many allergens possess epithelial modulatory activity and increase the permeability of this barrier [58]. Indeed, inhaled allergens can directly stimulate epithelial cells to produce a range of mediators such as thymic stromal lymphopoietin (TSLP) [59], IL-33 [60,61], and IL-25 [62]. These mediators can direct a polarised Th2 cell effector response and further perpetuate the salient features of asthma [63].

## 4 The inflammatory cascade—multi-cellular and fundamental to the pathogenesis of asthma

### 4.1 Inflammatory infiltrates

By virtue of their capacity to release a wide range of pro-inflammatory mediators, affinity for IgE and strategic proximity to blood vessels, mucosal surfaces and smooth muscle, mast cells have long been considered a key effector cell of asthma pathogenesis. Once synthesised, IgE antibodies circulate in the blood before binding to the high affinity Fcɛ antibody receptor (FcɛR)-I, present on tissue mast cells and peripheral blood basophils, or the lower affinity FcɛRII, found on a wide variety of leukocytes [64]. Mast cells are strategically located in both the airway epithelium and deeper layers of the mucosa [65]. In the asthmatic airways, the majority of IgE is bound by FcɛR1 on mast cells making them highly responsive to inhaled antigen [66]. Cross-linking of receptor bound IgE aggregations on the surface of these cells triggers a complex network of intracellular signalling events [66]. Leading to degranulation and discharge of preformed mediators such as proteases and vaso-active histamines, synthesis of lipid derived eicosanoids, leukotrienes (LTs) and prostaglandins (PGs), and the transcription of cytokines [67]. These mediators cause the early phase asthmatic reaction (EAR) which is directly responsible for AHR and is characterised by mucus secretion and vasodilatation prior to the recruitment of inflammatory cells [68]. The EAR lasts 30–60 min and followed 4–6 h later by the late phase asthmatic reaction (LAR) featuring excessive Th2 driven eosinophilic inflammation.

The importance of mast cells in atopic asthma became somewhat overshadowed by developments in the understanding of Th2 cell biology in asthma. However, recent developments in the field of mast cell biology have led to a resurgence of interest [69,70]. Mast cells can provide an early source of pro-inflammatory mediators such as IL-4, which can influence the proximal events during allergen-specific effector T cell sensitisation [67], and IL-5 which promotes eosinophil recruitment and activation [71]. Interestingly, the airway smooth muscle (ASM) bundles are infiltrated with mast cells in asthmatics [72]. The ASM is responsible for airway constriction and provides recruitment and survival factors for mast cells [72]. Interestingly microlocalisation of mast cells to ASM is absent in non-asthmatics eosinophilic bronchitis patients [73], which share many clinical manifestations with asthma such as eosinophilia and cough, yet demonstrate normal airway responsiveness [74]. This has led to the idea that selective mast cell microlocalisation within the lung structure may be a key factor in determining the declining lung function seen in asthmatics [73].

Granulocytic eosinophils typically predominate the atopic allergic inflammatory infiltrate [75], and the degree of eosinophilia has been correlated with disease severity in some asthmatic patients [76]. Eosinophils are found in both the lung tissue and the bronchoalveolar lavage (BAL) fluid of asthmatic patients [75,77], and are, in large, recruited from the bone marrow as CD34^+^ precursors following the release of prostaglandins, cysteinyl leukotrienes, cytokines and chemokines such as monocyte chemotactic protein (MCP)-1/CCL2, MIP3α/CCL20 and Regulated upon Activation, Normal T cell Expressed and Secreted (RANTES)/CCL5 from the asthmatic airway [78]. Selective recruitment of eosinophils to the airways is driven by Th2 mediated induction of the eotaxin family of chemoattractants (eotaxin-1/CCL11, eotaxin-2/CCL24 and eotaxin-3/CCL26) acting through the CCR3 chemokine receptor [79]. Eotaxin production is largely produced by epithelial cells in response to Th2 derived cytokines such as IL-13 and IL-5 [80]. A number of cytokines can prime eosinophil maturation including IL-3, and graulocyte–monocyte colony stimulating factor (GM-CSF) [78]. In particular, IL-5 is important for eosinophil recruitment [81], as well as the differentiation, proliferation and maturation of eosinophils [78].

Once recruited, primed eosinophils degranulate to release their cationic proteins including major basic protein (MBP) and eosinophil peroxidise (EPO) [82]. This capacity is thought to extend from the traditional role of eosinophils in protecting the host against parasitic worms [83]. These products are toxic to the lung microenvironment and, in addition to superoxide production, cause damage to the surrounding tissue microenvironment [78]. Inflammatory mediator release including Th2 cytokines (IL-4 and IL-13), and lipid eicosanoids (prostaglandins, leukotrienes and lipoxins), from eosinophils also contribute to the clinical symptoms of asthma through their potent effects on airway vascular tissue and smooth muscle reactivity [84]. Together, these products promote pathophysiological hallmarks of asthma [78]. Once established, the repetitive cycle of tissue damage and inflammatory-cell recruitment becomes chronic even in the absence of sustained allergen, the chronic inflammation persists [85]. Recent studies have also identified an immune modulatory role for eosinophils including antigen presentation function, attributing eosinophils the ability to direct effector the lymphocyte response [86]. In addition, eosinophils also possess the ability to synthesise, store and secrete the immunoregulatory IL-10 [87] and the Th1 cytokine IFN-γ which may skew the pathogenic Th2 response [88]. The importance of this during allergic asthma requires further attention.

Basophils are the least abundant granulocyte in the allergic airways and share many of their recruitment mechanisms with eosinophils thus often considered to be an accompaniment of T cell dependent allergic eosinophilia [89]. Lack of distinct functional markers has hindered understanding of their contribution to asthma pathogenesis, however basophils do express FcɛRI and contribute to the local symptoms of inflammation and AHR through degranulation and release of eicosanoids and histamines [90], and potentially via release of cytokines such as IL-4 [45], and APC function [46–48], which has led to a new appreciation of their role during both initiation and amplification of the allergic Th2 effector response [90].

The link between asthma and eosinophilic inflammation is long established [75]. Although eosinophilia is the most characteristic type of inflammation in asthma, this is neither an exclusive feature nor the only type of inflammation observed [91] and eosinophilic asthma is now considered a distinct phenotype of asthma associated pathologically, by thickening of the basement membrane, and pharmacologically, by corticosteroid responsiveness [92]. Elevated numbers of neutrophils and neutrophil-derived products without significant eosinophilia has been reported in the airways of asthmatics [93]. In particular, this has been associated with cases of severe asthma [10,94], asthma exacerbations [95] and particular asthma phenotypes such as non-atopic asthma [96]. It is important to note that neutrophilic asthma and eosinophilic asthma are not mutually exclusive and substantial overlap has been reported between each [92,97]. Indeed, recent studies have yielded new insights about the clinical and pathological correlates of eosinophilic and neutrophilic inflammation in asthma [98], and it is now recognised that non-eosinophilic asthma represents a sizeable subgroup of asthmatics [99]. This has reinforced the concept that asthma is a heterogeneous disorder which has several specific endotypes [9], that are associated with distinct clinical phenotypes and different inflammatory responses [97]. Importantly, this bears important considerations for response to therapy exemplified in non-eosinophilic asthmatics which demonstrate a poor response to inhaled corticosteroids, the mainstay of asthma therapy [100].

### 4.2 The Th2 lymphocyte response in the immunopathogenesis of asthma

One of the fundamental immunopathological features of allergic inflammation is the development of an allergen-specific effector Th2 response [20] and accompanying long-lived memory cells that continue to survey the airways for specific inhaled antigens [29]. Functional differentiation of Th effector subsets from naïve CD4^+^αβT cells involves the activation or repression of whole sets of genes resulting in differential expression of lineage defining transcription factors, effector mediator profiles and phenotypically distinguishing chemokine receptor expression which enables the subset to create a self-sustaining loop [101]. Until recently, differentiation into the mutually exclusive Th1 and Th2 effector cell subsets represented the sole paradigm of functional naïve CD4^+^T cell diversification [101,102]. Currently, several different effector fates for naïve CD4^+^Th cells have been identified and, in contrast to original thinking that Th effector differentiation was a terminal event [103,104], recent reports demonstrating functional plasticity within the Th2 subset suggests that this may not be the case [105]. Novel developments in the field of Th differentiation are likely to yield important information regarding our future understanding of the heterogeneity of chronic T cells in the design of therapeutics for chronic inflammatory disorders such as asthma.

Allergen-specific Th2 effector cells migrate to the lungs and enable the airways to recognise and respond to environmental allergens [106–108]. An increase in effector Th2 lymphocytes and their mediators has been reported in the bronchial mucosa and BAL fluid of asthmatic patients [107]. Elevated Th2 numbers also correlate with disease severity [107,109] and there is now overwhelming experimental and clinical evidence supporting the critical importance of Th2 cells during allergic sensitisation and propagation [106,108]. Phenotypically, Th2 cells are defined by expression of T1/ST2 [110,111] and the chemokine receptors CCR3 [112], CCR4 [113] and CCR8 [114]. The chemoattractant ligands for these Th2 associated receptors include eotaxin-1/CCL11, RANTES/CCL5, Thymus and Activation Regulated Chemokine (TARC)/CCL17 and Macrophage Derived Chemokine (MDC)/CCL22 are upregulated in the allergic airway [115,116]. The potential of Th2 cells to promote allergic immunopathology is amplified by production of a range of mediators including IL-4, IL-5, IL-9, IL-13 and IL-25 which together promote the salient feature of asthma such as IgE production, AHR, inflammation and tissue remodelling [117,118]. IL-4 and IL-13 are involved in the class switching of B cells to IgE synthesis [119], and favor mucus secretion and fibrosis [120,121]. Th2 cytokines also activate secondary effector cells in asthma including the recruitment of mast cells (IL-4, IL-9 and IL-13) [67] and basophils (IL-3 and IL-4) [90], whilst IL-5 supports growth, differentiation, and activation of eosinophils [78].

**Fig. 3:**
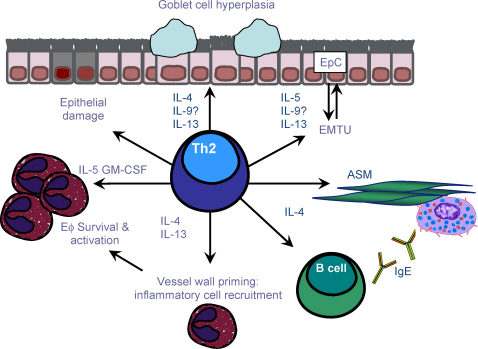
Th2 effector cells and asthma pathogenesis. Th2 cells have a central role in orchestrating the allergen-induced inflammatory response. Th2 derived IL-4 and IL-13 stimulate B cells to synthesise IgE whilst IL-5 is necessary for eosinophilic inflammation. Th2 cytokines are also involved in mast cell proliferation and allergic airway remodelling. *Key*: Eϕ, eosinophil; EpC, epithelial cell; EMTU, epithelial to mesenchymal tropic unit; ASM, airway smooth muscle; AHR, airway hyperreactivity.

IL-25, also known as IL-17E, is a product of activated Th2 cells and innate cells such as mast cells. Evidence suggests that IL-25 enhances antigen induced Th2 cytokine production and eosinophil recruitment to the airways [122]. IL-25 mediated enhancement of antigen induced eosinophil recruitment can be inhibited by the depletion of CD4^+^T cells suggesting this mediator promotes allergic airway inflammation by a CD4^+^T cell dependent mechanism [122]. Although originally thought to be a Th2 derived cytokines, the Th cell source of IL-9 in the allergic airway requires clarification in light of the recent identification of an IL-9 producing Th9 subset [105,123]. An overview of the main immunopathological features mediated by the Th2 response in the allergic airway is given in Fig. 3.

### 4.3 Emerging T lymphocytes in the immunopathogenesis of asthma

Although the asthmatic response is considered to be dominated by Th2 lymphocytes, it is now clear that there are assorted layers of complex underlying T cell mediated responses present in the allergic lung [124], and it is now thought that asthma may not solely be controlled by the Th2 subset [125]. The immunopathological potential of these different lymphocyte types and the potential synergy between them must be considered when studying complicated inflammatory diseases such as asthma. This is discussed further below.

Th1 cells preferentially produce the mediators IFN-γ, IL-2, TNF-α and lymphotoxin, activate potent microbicidal activity from macrophages and antagonise the development of Th2 responses [113,126,127]. IFN-γ producing Th1 cells have been documented in the airways and serum of asthmatic patients however their contribution to asthma pathogenesis is not clear [107,128]. T-bet, the Th1 defining transcription factor is under expressed in the asthmatic airway [129] and T-bet^−/−^ mice have severe defects in Th1 cell differentiation and susceptibility to Th2 biased asthma-like disease [130], suggesting a regulatory contribution. Moreover, addition of Th1 related cytokines has been demonstrated to inhibit allergic airway inflammation by inhibiting the Th2 response *in vivo* [131–133]. Conversely, introduction of Th1 cells into a murine asthma model was reported to worsen airway inflammation suggesting they may cause further lung immunopathology [134].

An IL-17 producing effector fate for naïve CD4^+^Th cells in the presence of TGF-β with either IL-6 or IL-21 has recently been described [135,136]. These “Th17” cells express the IL-23 receptor (IL-23R) [137,138], are characterised by production of IL-17 (IL-17A), IL-17F and IL-22 [135,139,140], and are now acknowledged as a distinct lineage from Th1 and Th2 cells [141–143]. IL-17 acts on a broad range of leukocytes and has been found at elevated levels in the airways of asthmatics however the functional relevance to this to the asthmatic disease state remains unclear [144,145]. IL-17 is thought to be critically involved in the proximal events during allergen sensitivity [146]. Conversely, it has been demonstrated that IL-17 has a negative effect on the established allergic airway disease [146] and an inhibitory influence on the production of pro-asthmatic mediators from fibroblasts [147]. To date, studies on Th17 cells in the allergic lung have used models of Th2 adjuvant driven allergic pathology; however, the effect of Th2 skewing adjuvant compounds on the development and maintenance of Th17 responses is not known. Chronic asthmatics can develop steroid resistance which is difficult to treat [148]. Th17 cells, but not Th2 cells, have been demonstrated to be mediate steroid resistant asthma *in vivo* [149]. Much more research is required to further understanding of the biology of this Th lineage and fully address their role in chronic inflammatory diseases such as asthma.

Allergen-specific CD8^+^T cells have also been observed in asthmatic patients [108,150,151]. Exploration of whether CD8^+^T cells contribute to or suppress the allergic phenotype *in vivo* has been inconclusive. Reduced AHR, eosinophilic inflammation and IL-13 production has been reported mice deficient in CD8^+^T cells, restorable upon reconstitution of these cells [152,153]. Conversely, depletion of CD8^+^T cells during allergic sensitisation enhanced Th2 responses suggesting their involvement in asthma pathogenesis may be stage dependent [154]. This pro-allergic capability has been related to their ability to produce significant amounts of IL-5, the critical effector in asthmatic eosinophilia [155,156]. Indeed, transfer of IL-5 producing CD8^+^T cells induced eosinophilic inflammation in the lung following allergen challenge [157]. Allergen-specific CD8 T cells have also shown suppressive activity *in vivo* through production of Th1 skewing IL-12 [158]. This information suggests CD8^+^T cells have the potential to modulate the course and outcome of allergic disease.

### 4.4 Non-classical lymphocytes in the immunopathogenesis of asthma

Although most immune cells are classified as either innate or adaptive, unique non-classical lymphocytes such as γδT cells [159], NK cells [160], and NKT cells [161], possess characteristics of both the innate and adaptive immune system. Despite the established role of allergen-specific antibodies [64], and effector and memory αβT cell responses [162] in allergic immunopathology, the importance of non-classical innate-like lymphocytes in the allergic airway has recently been the focus of asthma research [163,164]. Natural killer (NK) cells are normally present in the lung and express a variety of receptors which enable a primary immunosurveillance function [160]. Increased numbers of NK cells and NK cell activity have been observed in patients with asthma but little is known of their role in the allergic airway allergens [165,166]. The contribution of NK cells to allergic airway disease has been studied *in vivo* using OVA sensitisation/challenge systems [167]. Depletion of NK cells before immunisation or during challenge was reported to inhibit pulmonary eosinophilia, T cell infiltration and associated inflammatory mediators suggesting a pathological contribution to both development and maintenance of allergic airway disease [167].

### 4.5 iNKT cells

Natural killer T cells (NKT cells) are innate lymphocytes expressing a highly conserved TCR and innate-like receptors characteristic of NK cells [168]. iNKT cells are selected and restricted by the non-classical MHC I-like CD1d molecule [169] recognising endogenous and exogenous glycolipids such as α-galactosyl ceramide (αGC) [170]. Direct activation of NKT cells by glycolipids results in the production of large amounts of Th1 and Th2 cytokines such as IFN-γ and IL-4 [161]. This function attributes them potent immune immunopotentiating effects prior to development of adaptive immune [171,172]. iNKT cells are found in the lungs and increase in response to allergen challenge; however, the role of iNKT cells in the allergic lung has been controversial [171,173]. *In vivo* murine studies have demonstrated a contributing role to allergic AHR and airway inflammation independent of allergen priming [164,174,175]. Rapid Th2 cytokine release is thought to promote the development of allergic Th2 responses. In addition, a subset of iNKT cells preferentially producing IL-13 and expressing the receptor for pro-allergic cytokine IL-25 has recently been demonstrated and may potentiate IL-25 induced propagation of allergic airway inflammation [176]. Jin et al., used an allergic model of OVA sensitisation/challenge model to demonstrate that Vγ1^+^AHR-promoting γδT cells and iNKT cells synergise in the development of AHR and that they depend on each other for this function [177]. In addition to *in vivo* studies indicating a role for NKT cells in the development of allergen-induced AHR, Akbari et al., reported that greater than 60% of CD4^+^T cells in the airways of moderate–severe asthmatic patients were NKT cells [171]. Subsequently, studies demonstrating that NKT cells were a minority population [173]. In support of these findings, Thomas et al. found similarly low numbers of invariant natural killer T cells in subjects with asthma not receiving corticosteroids [178]. The start differences in these studies have since been attributed to the difficulty in detection and analysis of these cells [179,180]. Further work is required to clarify understanding of iNKT cells in allergic asthma.

### 4.6 γδT cells

Although both conventional αβ and γδT cells arise from thymic progenitors, γδT cells share many of their signatory properties with innate leukocytes residing predominately in the alveoli epithelium, lamina propria, smooth muscle layers and around blood vessels walls where they make frequent contact with the more numerous resident leukocytes [181,182]. An increase in pulmonary γδT cells has been observed in asthmatic patients, in particular during symptoms of acute asthma exacerbations [183–185]. Although their role in the allergic airway is not yet clear, a great deal of recent interest has focussed on their ability to modulate adaptive immune responses, specifically their role in normal airway function and lung homeostasis [181,186]. Recently, an important role for γδT as regulators of normal airway tone has recently emerged [187] Increased AHR in allergen sensitised and challenged γδT cell deficient (TCRδ^−/−^) or depleted mice has been observed [188,189]. Additionally, non-sensitised γδT cell deficient mice receiving allergen challenge demonstrated increased AHR with no accompanying overt inflammatory changes [188]. Interestingly, negative regulation of AHR by γδT cells was found to be independent of αβTh2 driven inflammation and eosinophilia [188]. How γδT cells exert their effects on airway tone is not defined at present. γδT cells do not appear to recognise allergen directly [190], and the regulation of airway responsiveness by γδT cells is not antigen specific [177]. Potential targets including direct interaction with ASM, mast cells or neuronal components that regulate airway tone have been suggested [188]. The release of mediators triggered by a stress reaction or modulation through epithelial repair or innate leukocytes has also been proposed. Early studies by McMenamin et al. demonstrated suppression of the allergic αβTh2 driven response by an anti-inflammatory γδT cell population elicited in response to repeated exposure to aerosolised antigen [191]. However, there are now several contrasting studies which support the notion that γδT cells potentiate the activation of allergic Th2 inflammatory responses [163,188,192,193]. The conflicting opinions on whether γδT cells are anti- or pro-inflammatory in allergic inflammation have in part been resolved by consideration of the differences in *in vivo* systems and experimental programmes [186].

## 5 Regulation and resolution of allergic inflammation

The majority of research into asthma has focussed on elucidating the pro-inflammatory pathways underlying disease pathogenesis and consequently many of the current therapeutic targets focus on blocking the initiating or amplifying components [194]. Paradoxically, the necessity of appropriate termination and resolution of inflammation has comparatively recently been recognised [195]. This has led to the concept that chronic inflammation may arise as a result of a lack of specific ‘stop’ signals for inflammatory responses. Coupled with continued presence of allergens, this might explain the chronic nature of allergic disease. Natural resolution of inflammation is a dynamic process which requires the removal of stimulus, down regulation of mediators and elimination of dead cells [195]. Dysregulation or failure of this process prevents a return to homeostasis and can contribute to the pathogenesis and progression of chronic inflammatory disorders such as asthma [196]. Many of the endogenous, pro-resolving and anti-inflammatory mechanisms which co-ordinate the resolution process have begun to be defined and are thought to originate from the inflammatory response [197,197].

### 5.1 Regulatory processes

The removal of apoptotic cells at the termination of inflammation (termed efferocytosis) promotes a return to homeostasis. In addition to cell clearance, efferocytosis results in collateral release of anti-inflammatory mediators such as IL-10 which contribute to restorations of tissue homeostasis and growth factors which may additionally aid maintenance of lung tissue integrity [198]. An increase in apoptotic cells has been observed in the asthmatic lung and it has been proposed that efferocytosis may be impaired in such situations [199,200]. Therapeutic approaches to promote clearance of inflammatory cells from inflamed sites may reduce host damage during inflammatory disease such as asthma.

Altered chemokine production is a feature of allergic airway inflammation and responsible for inflammatory leukocyte recruitment and retention within the lungs [115]. As inflammation resolves, chemokines need to be removed from the microenvironment to halt further leukocyte recruitment. The “silent” chemokine receptor D6 plays an important role in scavenging inflammatory, but not constitutive chemokines [201]. D6 knockout mice fail to resolve acute inflammatory responses, suggesting that D6 has important roles in resolution [202]. Infiltrating leukocytes can also upregulate selected chemokine receptors as they undergo apoptosis, in order to sequester chemokines and so prevent further leukocyte recruitment [203].

A novel genus of local acting anti-inflammatory lipid mediators with pro-resolving and protective properties, coined resolvins and protectins, are produced from omega-3 fatty acids (EPA and DHA) [197,204] and are actively synthesised during resolution of inflammation. These molecules function to stop granulocyte infiltration, prevent pro-inflammatory chemokine release and activation of inflammatory cells whilst stimulating clearance of inflammatory cells controlling the duration and magnitude of inflammation. Resolvin E1 (RvE1) acts as an anti-inflammatory and pro-resolution lipid mediator of allergic airway inflammation [205,206]. These recent studies have revealed important connections between lipid biology and immunology. Further understanding of these pathways is likely to expand therapeutic intervention possibilities.

### 5.2 Regulatory T cells

CD4^+^ regulatory T lymphocytes (Tregs) play a key role in control of inflammatory responses [207]. These cells can be naturally occurring (nTreg), selected by high avidity interactions in the thymus, or induced (iTreg) extrathymically from naïve conventional CD4+T cells under subimmunogenic antigen presentation, during chronic inflammation and during homeostasis [208]. Tregs constitutively express high levels of the IL-2 receptor-α chain (CD25) and are maintained by TGF-β. The transcription factor, Forkhead box protein-3 (FoxP3) is important to generation of CD4^+^CD25^+^FoxP3^+^Tregs and also used as an identification marker [209,210]. An important role for CD4^+^CD25^+^FoxP3^+^Tregs to immune regulation of a variety of chronic inflammatory disease scenarios including asthma has been noted [211]. Treg function is thought to be impaired in allergic patients since CD4^+^CD25^+^FoxP3^+^Tregs from non-allergic donors but not allergic donors suppressed proliferation and Th2 cytokine secretion by CD4^+^CD25^−^T cells [212]. A direct link between CD4^+^CD25^+^FoxP3^+^Tregs and suppression of AHR in asthma has now been demonstrated *in vivo* [213]. In a chronic model of allergic airway disease, transfer of antigen-specific Tregs suppressed features of established allergic airway inflammation and prevented the development of airway remodelling [214]. These data suggest that exploiting the regulatory properties of CD4^+^CD25^+^FoxP3^+^Tregs *in vivo* could regulate asthmatic disease. Tregs elicit regulatory function by a variety of mechanisms including modulation of APC function [215] and induction of a state of non-responsiveness or ‘anergy’ in effector cells through secretion of immunosuppressive cytokines known as “bystander suppression” [216].

### 5.3 Regulatory cytokines

IL-10 is an important homeostatic cytokine in the airways [217] and has been reported to modulate many effector functions that are associated with allergic asthma including Th2 activation, IgE production [218], eosinophil and mast cell function [219–221] and inhibition of mast cell function [222,223]. Furthermore, diminishing IL-10 can break tolerance to allergens suggesting a role in regulation of responses to allergens [217]. Allergen-specific IL-10 producing Tregs were found to be significantly lower than healthy controls, suggesting that a defect in IL-10 production may contribute to the asthmatic phenotype [224]. *In vivo*, resolution of allergic airway inflammation by CD4^+^CD25^+^Tregs has recently been shown to be IL-10 dependent [225].

Although considered to be a pro-fibrotic cytokine, TGF-β also has potent anti-inflammatory activity, can inhibit T cell proliferation and differentiation and impair Ig synthesis and secretion [226]. TGF-β expression is upregulated in the lungs of asthmatic patients [227], and active TGF-β signalling is increased in allergen-induced airways disease in mouse [228], suggesting that TGF-β may play an important role in the inflammatory process. The implication that TGF-β may regulate the allergic inflammatory response in the airways has been investigated *in vivo* [229–231]. However, the pleiotropic nature of the cytokine however has consequently made it most difficult to dissect the pro or anti-inflammatory effects.

Exploiting the body's own endogenous counter-regulatory mechanisms offers therapeutic potential for common inflammatory diseases such as asthma. Further progress in this area is likely to prove important in the discovery of new and safe targets.

## 6 Airway remodelling

**Fig. 4:**
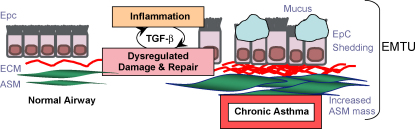
Airway remodelling in asthma. Activation of airway epithelium by aeroallergens and pollutants leads to downstream effects including inflammation, dysregulated repair, activated EMTU and tissue remodelling. *Key*: ASM, airway smooth muscle; ECM, extracellular matrix; EMTU, epithelial to mesenchymal trophic unit; Epc, EpC, epithelial cell; TGF-β, transforming growth factor-β.

Chronic inflammation is often accompanied by remodelling of the tissues and there is evidence of structural alterations to the airway architecture in asthmatics [232], which is collectively known as remodelling [233,234]. Airway calibre is determined dynamically by the constricting forces of ASM and elasticity and hysteresis of the parenchyma [235]. Remodelling changes contribute a thickening of the airway wall [236], and reduction of the airway diameter [237] which is believed to result in fixed airflow obstruction, persistent AHR and a poor short-term treatment response [238]. Remodelling changes include a thickened reticular basement membrane, dysregulated extracellular matrix (ECM) protein deposition and increased vasculature [239]. The remodelled lung also features striking increase in ASM mass with evidence for both hyperplasic and hypertrophic changes [240,241], in addition to recruitment of ASM progenitors [18,242]. Furthermore, the remodelled lung features compromised epithelial barrier function [243,244], and is accompanied by mucus gland hyperplasia [233]. A summary of these changes is depicted schematically in Fig. 4.

There is an increasing recognition that alterations to ECM composition play an important role in the remodelling process [245]. Mesenchyme derived myofibroblasts which have both fibroblast and ASM-like properties and a highly proliferative, contractile and secretory phenotype, are normally responsible for tissue repair after injury [246]. However, subepithelial myofibroblasts are increased in asthma and this has been correlated to basement membrane thickness in asthmatic patients [247,248]. Although they are considered key participants in airway remodelling [246], the mechanisms underlying myofibroblast induction are not fully understood. It has been proposed that fibroblast-like progenitors (fibrocytes) migrate to the allergic lung contributing to the myofibroblasts response [249]. In addition, ASM and fibroblasts can also develop a myofibroblasts-like phenotype in the remodelled lung [250–252]. Collectively, these processes are critical to the generation and regulation of ECM during airway remodelling [253–255]. Interestingly, loss of epithelial characteristics and subsequent acquisition of mesenchymal characteristics giving rise to fibroblasts and myofibroblasts has also been postulated [256]. This is known as epithelial–mesenchymal transition (EMT) [256]. The extent to which EMT occurs in the allergic lung has been the subject of active investigation [257,258]. Epithelial to mesenchymal cross talk propagates chronic allergic inflammation and provides the optimal environment for development of remodelling [11,259]. This interaction between epithelium and underlying mesenchyme in the asthmatic lung is thought to represent a reactivation of the developmental epithelial–mesenchymal-trophic unit (EMTU) associated with morphogenesis during foetal lung development [259]. It is proposed that allergen-induced epithelial damage and Th2 mediated inflammatory insult cooperate to promote functional disturbance of the EMTU [260].

### 6.1 Transforming growth factor-β: a key player in airway remodelling

The pleiotropic mediator TGF-β is produced by both immune leukocytes and structural cells and has potent pro-fibrotic activity [261]. TGF-β is upregulated in the airways of asthmatics [227], and is considered the master regulator of remodelling [262]. The TGF-β receptor is widely expressed and receptor-ligand signalling promotes ECM synthesis and myofibroblast transformation from ASM and fibroblasts [246,263]. TGF-β also propagates remodelling through a variety of other means including the induction of inflammatory mediators by structural cells (Fig. 3) [264], and has a direct effect on ASM contractility which can influence airway responsiveness [265,266]. *In vitro* studies have observed constitutive activation of the TGF-β signalling pathways in the allergic airways [227,228]. The importance of TGF-β as a pro-remodelling cytokine has also been illustrated *in vivo* using mouse models of asthma [267]. McMillan et al. demonstrated a reduction in peri-bronchiolar ECM deposition and decreased ASM proliferation following TGF-β blockade during established disease in a mouse asthma model [267]. TGF-β induced remodelling was also evaluated in mice lacking the TGF-β signalling component Smad-3. Following induction of allergic airway disease decreases in airway fibrosis were associated with a reduction in myofibroblasts without associated changes in inflammation [268]. Furthermore, overexpression of TGF-β in the lung induced severe fibrosis with characteristic remodelling ECM deposition [262,269].

In addition to TGF-β, additional mediators such as growth factors and proteinases released from inflammatory and structural cells in the airway create a complex environment that contributes to airway remodelling. Increased platelet derived growth factor derived growth factor (PDGF) is found in the asthmatic lung and promotes proliferation of fibroblasts and ASM [270,271]. Increased angiogenesis, and the pro-angiogenic cytokine Vascular Endothelial Growth Factor (VEGF) and its receptors have been noted in the asthmatic airway [272,273]. Furthermore, dysregulated production of extracellular matrix metalloproteinase (MMPs) family members has been reported in the asthmatic airway [274]. These proteinases are responsible for the degradation of the extracellular matrix during tissue remodelling [275].

### 6.2 Other important contributors to airway remodelling

There is now substantial evidence recognising the importance of eosinophils in airway remodelling [276]. Indeed, many eosinophil products are capable of influencing the differentiation, proliferation and function of lung structural cells [78,277]. Allergen sensitised mice can be induced to develop remodelling when exposed to prolonged allergen challenge regimens [251]. Sensitised Δdbl-GATA mice, which are devoid of eosinophils, subjected to a prolonged allergen challenge regimen, were significantly protected from remodelling [278]. Production of the pro-fibrotic mediator TGF-β by eosinophils is frequently observed in the lungs of asthmatics [279,280], and this is thought to be the predominant mechanism by which eosinophils fuel the development of remodelling [281]. This is supported experimentally, whereby IL-5^−/−^ mice, which have significantly reduced allergen-induced airway eosinophilia, are protected from TGF-β dependent remodelling [282,283], and clinically following anti-IL-5 treatment in asthmatic patients which reduced ECM deposition [280]. Additionally, eosinophils contribute to remodelling indirectly through TNF-β mediated induction of additional fibrotic factors such as plasminogen activator inhibitor-1 (PAI-1) and MMPs [284]. Neutrophils can deliver multiple granule products and reactive oxygen intermediates which promote inflammation and have tissue damaging potential which may contribute to airway remodelling [285,286]. Mast cells have a hyperplastic effect on ASM mass and induce collagen secretion from surrounding fibroblasts and myofibroblasts which may contribute to airway remodelling [287,288]. ASM derived chemokines can further recruit mast cells and promote their degranulation establishing a positive feedback loop in the allergic lung [289,290]. Furthermore, mast cells are a source of pro-fibrotic TGF-β [68] and have also been shown to produce matrix active MMPs in the allergic lung [291].

## 7 Airway inflammation and remodelling: parallel or sequential events

It has generally been accepted that remodelling occurs as a consequence of prolonged cycles of damage and repair as a consequence of Th2 led immunopathology. However, despite the progress that has been made regarding airway remodelling, several critical questions remain unresolved. This has been excellently reviewed elsewhere [117,233,292]. One particular controversy is whether chronic inflammation and remodelling progress as parallel or sequential events. Reports of allergen-induced structural changes in the airway prior to symptomatic asthma [293], imply that remodelling occurs early. Furthermore, remodelling in established asthma is poorly responsive to current therapies, such as inhalation of corticosteroids and administration of beta(2)-agonists, anti-leukotrienes [294], and cannot account for the heterogeneity of human asthma phenotypes. More recent theories propose that dysregulated injury/repair processes stemming from the susceptibility of the bronchial epithelia to components of the inhaled environment might precede, or occur in parallel with airway inflammation. This hypothesis is strongly supported by paediatric studies [295,296], and the concept that the EMTU is abnormally sensitive to environmental factors due to a genetic or prenatal environmental basis and as a result becomes abnormally activated [297]. This may help explain why the prolonged use of anti-inflammatory therapy does not always correlate with the natural history of asthma. Improvements to *in vivo* systems in which to study remodelling will be imperative to the understanding of this question.

### 7.1 Future considerations for airway remodelling

Airway remodelling is a complex and dynamic process that is now considered to be fundamental to the chronicity of the asthmatic disorder [298]. Although the function implications of these structural changes are only beginning to be understood, it is thought that remodelling can have serious repercussions for the response to therapy and can contribute to airway obstruction [15,299]. Indeed, a correlation between airway wall thickening and a decline in airway function of asthmatic patients has been reported [300]. Current asthma therapies such as inhaled corticosteroids are directed towards reducing inflammation and have only a partial effect on remodelling [301]. This has profound problems for the future of disease management [302]. Controversially, associations between airway remodelling and asthma severity have been inconsistent and it cannot be excluded that thickening of the airway wall provides functional protection against airway narrowing in asthma [233]. A better understanding of the mechanisms underlying airway remodelling, its reversibility/prevention and its complex relationship with airway inflammation is a prerequisite for developing therapeutic.

## 8 Future challenges and prospects for asthma therapy

*In vitro* and *in vivo* asthma models and clinical studies have disclosed much information regarding asthma pathogenesis including many targets amenable to therapeutic intervention. However, there is still no cure for asthma and progress in the development of effective new compounds for asthma therapy has been comparatively slow. The cornerstones of current asthma therapy (β(2)-agonists and inhaled corticosteroids) provide symptomatic relief and some physiological improvements for most asthma sufferers [148,303]. Whilst this approach has been reasonably effective, incidences of steroid resistant asthma is a major drawback and the side effects of steroid therapy can lead to poor patient compliance [304]. Furthermore, asthma symptoms return as soon as corticosteroid therapy is withdrawn [305]. Consequently, there is a need for more tailored and specific therapies that target the local pathways involved in asthma pathogenesis.

Several recent and important discoveries in Th cell biology, self-limitation of inflammation and immune regulation carry therapeutic potential as strategy for asthma treatment. Understanding and optimising the body's own endogenous regulatory and pro-resolving local inhibitory mechanisms system offers a potentially safe and effective approach for tailored development of asthma therapy, in particular acute inflammatory events and steroid refractory allergic inflammation which is otherwise difficult to treat. The immune system comprises complex, multi-cellular layers that are tightly regulated and function with the surrounding microenvironment to maintain homeostasis and provide host defence. Cells with regulatory potential such as CD4^+^CD25^+^FoxP3Tregs and γδT cells are also an attractive target for asthma therapy. To harness the therapeutic potential of the immune system, a variety of factors need to be considered including mode of activation, migration and function. By identifying and understanding these interactions it will be possible to further define their benefit to allergic airway responses without losing the ability to fight infection. This knowledge is essential for development of immune modulatory intervention strategies and fine tuning the host response to maximise the protective and minimise the destructive aspects of the host allergic response. Consideration must also be given to the contribution of different Th cell subsets and the complicated and largely undefined relationship between them. Further understanding of these components will be vital to identification of potential mechanisms to tip the balance between regulatory and pro-allergic immune responses. Furthermore, understanding of regulatory pathways and the relationship between inflammation and remodelling may aid the development of new strategies for the chronically remodelled lung environment which is refractory to anti-inflammatory treatments and also the development of structural alterations seen in childhood which occur independent of inflammation or prior to overt symptoms.

### 8.1 A new perspective on asthma pathogenesis

Asthma is an extremely complex disease. It is now clear that asthma is not solely determined by a Th2 response but instead reflects a constantly changing immune response that features complex counter regulatory and effector networks between the microenvironment of structural cells in the context of the cellular leukocytes. Innate-like lymphocytes represent a common pathway through which a number of potential mechanisms operate during inflammatory regulation. Identifying defects in this pathway could facilitate the diagnosis of asthma, and could be used to monitor asthma control and hence be useful in clinical decision-making.

The conceptual lines between innate and adaptive immunity have been clearly drawn in current dogma of the immune system. Adaptive immunity is thought to develop during the lifetime of an individual, mediated by selectable receptors. In contrast innate immunity is common to an entire species and hardly modified by an individual's history. As more becomes known of the plasticity within the innate and adaptive immune system, current theoretical concepts must be constantly re-evaluated. With this, the lines between innate and adaptive pathways become less distinct. Current understanding of the interface between innate and adaptive immunity, leukocytes which share properties with both arms of the immune system, and the immune modulatory potential of structural cells in the airways is continually growing. Arguably, deeper understanding of these relationships will no doubt continue to add an extra twist to the already complicated immune network and regulatory pathways underlying chronic inflammatory disease.

## Conflict of interest statement

The authors have no conflicting interest.
